# Supplementary figures and images for: Compound 21, a two-edged sword with both DREADD-selective and off-target outcomes in rats
Source: PLoS One. 2020 Sep 18;15(9):e0238156. doi: 10.1371/journal.pone.0238156 (PMC7500623; doi:10.1371/journal.pone.0238156)

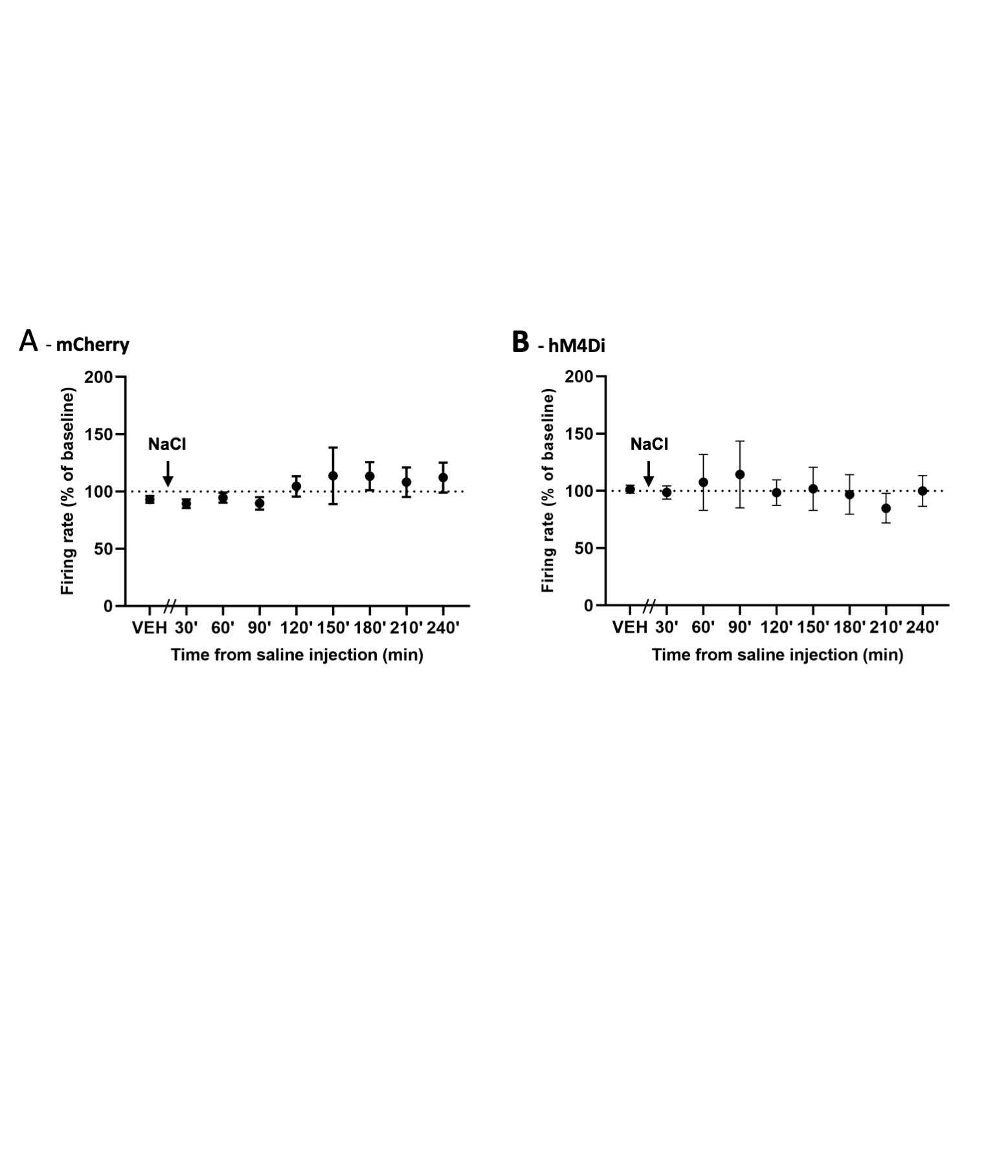

Supplement: S1 Fig — Effect of vehicle along time on SNc neuronal activity rate in rats expressing mCherry (n = 8 recordings, 5 animals) (A) or hM4Di (n = 8 recordings, 5 animals) (B). To keep the same experimental conditions, two saline injections were realized, one at the end of the 10-minutes baseline recording (VEH, a 20-minutes interval) and one at the end of the vehicle period corresponding to the time of C21 injection (30-minutes intervals). Data were expressed as the mean number of recording sides +/- SEM. (TIF) [file pone.0238156.s001.tif]

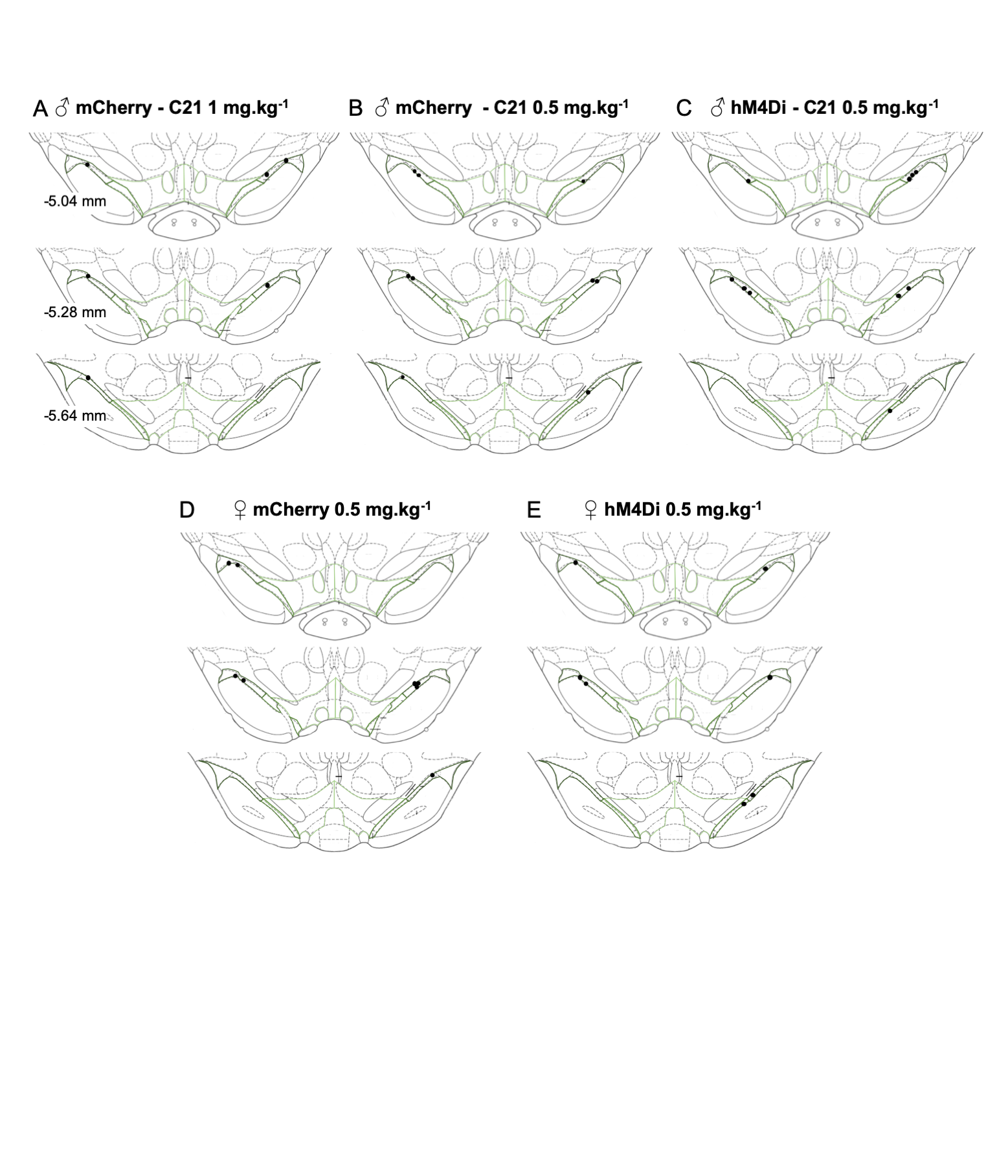

Supplement: S2 Fig — Male expressing mCherry treated with 1 mg.kg-1 of C21 (A) or 0.5 mg.kg-1 of C21 (B). Male expressing hM4Di treated with 0.5 mg.kg-1 of C21 (C). Female expressing mCherry (D) or hM4Di (E) and treated with 0.5 mg.kg-1 of C21. (TIF) [file pone.0238156.s002.tif]

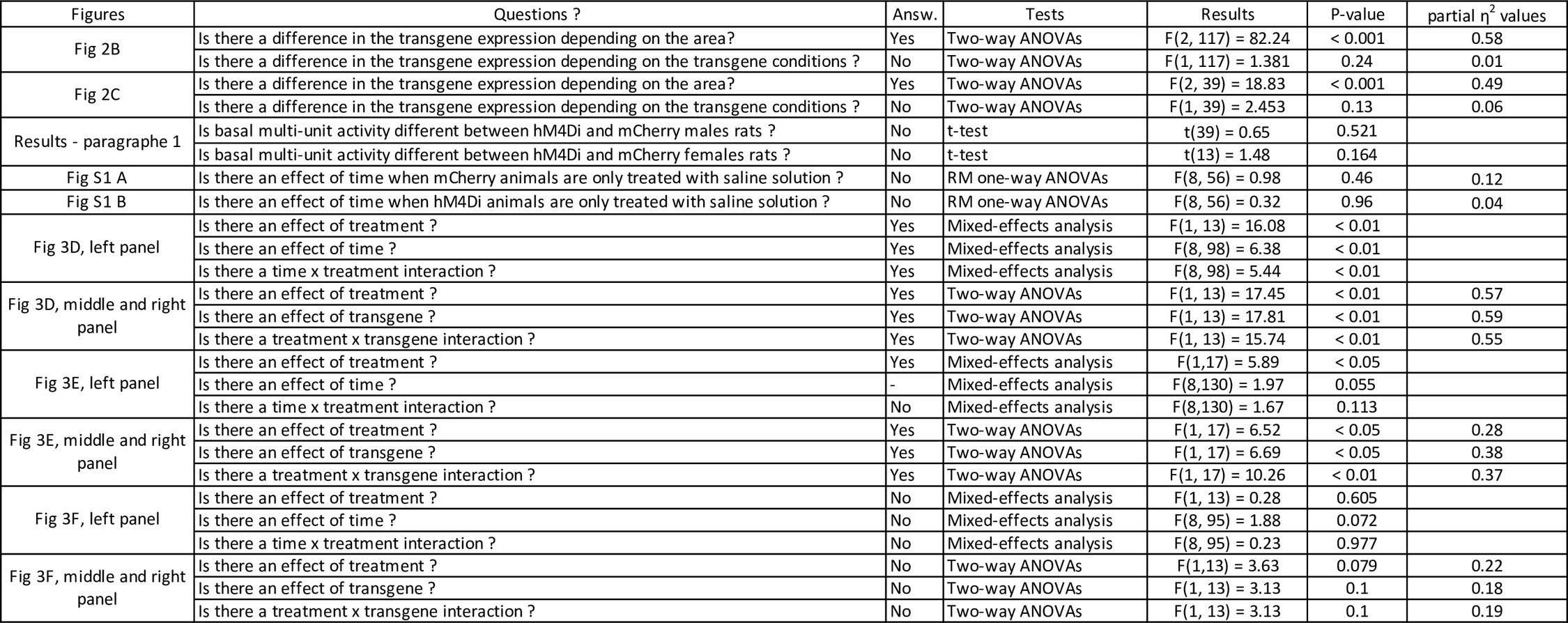

Supplement: S1 Table — (TIF) [file pone.0238156.s003.tif]
